# Arachnids Secrete a Fluid over Their Adhesive Pads

**DOI:** 10.1371/journal.pone.0020485

**Published:** 2011-05-26

**Authors:** Anne M. Peattie, Jan-Henning Dirks, Sérgio Henriques, Walter Federle

**Affiliations:** 1 Department of Zoology, University of Cambridge, Cambridge, United Kingdom; 2 Department of Biology, University of Évora, Évora, Portugal; Field Museum of Natural History, United States of America

## Abstract

**Background:**

Many arachnids possess adhesive pads on their feet that help them climb smooth surfaces and capture prey. Spider and gecko adhesives have converged on a branched, hairy structure, which theoretically allows them to adhere solely by dry (solid-solid) intermolecular interactions. Indeed, the consensus in the literature is that spiders and their smooth-padded relatives, the solifugids, adhere without the aid of a secretion.

**Methodology and Principal Findings:**

We investigated the adhesive contact zone of living spiders, solifugids and mites using interference reflection microscopy, which allows the detection of thin liquid films. Like insects, all the arachnids we studied left behind hydrophobic fluid footprints on glass (mean refractive index: 1.48–1.50; contact angle: 3.7–11.2°). Fluid was not always secreted continuously, suggesting that pads can function in both wet and dry modes. We measured the attachment forces of single adhesive setae from tarantulas (*Grammostola rosea*) by attaching them to a bending beam with a known spring constant and filming the resulting deflection. Individual spider setae showed a lower static friction at rest (26%±2.8 SE of the peak friction) than single gecko setae (*Thecadactylus rapicauda*; 96%±1.7 SE). This may be explained by the fact that spider setae continued to release fluid after isolation from the animal, lubricating the contact zone.

**Significance:**

This finding implies that tarsal secretions occur within all major groups of terrestrial arthropods with adhesive pads. The presence of liquid in an adhesive contact zone has important consequences for attachment performance, improving adhesion to rough surfaces and introducing rate-dependent effects. Our results leave geckos and anoles as the only known representatives of truly dry adhesive pads in nature. Engineers seeking biological inspiration for synthetic adhesives should consider whether model species with fluid secretions are appropriate to their design goals.

## Introduction

Climbing animals use a vast array of attachment strategies to scale vertical and inverted surfaces [1]. Adhesive footpads, found among arthropods, amphibians, reptiles and mammals, allow strong, repeatable attachment to both smooth and rough, hard and soft substrates [2]. Geckos and anoles adhere by dry, intermolecular adhesion [3], [4], but other organisms secrete a fluid over their adhesive pads, including insects [5]–[9], frogs [10] and bats [11]. Pad secretions are thought to enhance attachment forces by contributing capillary and viscous adhesion, and increasing overall contact on rough substrates [6], [12]. Although fluids are frequently used in industry to lubricate solid-solid interfaces, wetted insect adhesive pads can resist significant shear forces during climbing [5], [6], [13].

**Figure 1:**
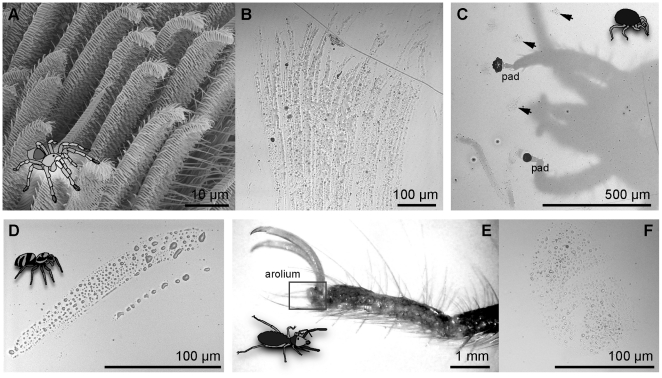
Arachnids investigated for this study. (A) Tarantula (*Grammostola rosea*) setae. (B) Fluid trail left behind by a *Grammostola* tarsus. (C) Mite (*Gromphadorholaelaps schaeferi*) clinging upside down to a polystyrene-coated glass coverslip, showing two adhesive pads in contact. Footprints are indicated by arrowheads. A trail of fluid is also visible, lower left. (D) Jumping spider (*Salticus scenicus*) fluid trail from one tarsus. (E) Solifugid (*Gluvia dorsalis*) tarsus, arolium situated distally, at base of claws. (F) Fluid footprint left by one *Gluvia* arolium.

Arachnids have attracted little attention from researchers in the field of biological adhesion, despite representing a wide diversity of adhesive morphologies. Some mites have hairy, or “fibrillar”, adhesive pads [14] while others have smooth ones [15]. Amblypygids, pseudoscorpions and solifugids are all known to bear smooth pads on their feet and pedipalps [16]–[18]. Spider adhesive pads most closely resemble the fibrillar pads of geckos, with large arrays of branched hairs (“setae”) terminating in flattened tips, called “spatulae” (Fig. 1a; [19]–[21]). We would predict that the very small size of the spiders' spatulae (200–300 nm wide; [22]) allows them to make close contact with the substrate, attaching without the aid of a fluid as the gecko does [3], [4], [23]. Indeed, this has been the consensus among researchers who have studied spider adhesion [19]–[21], [24], [25].

Here we investigate the adhesive feet of selected arachnids (spiders, mites and solifugids) for the presence of fluid secretions. We used two independent approaches. First, we imaged the footpad-substrate interface directly with interference reflection microscopy (IRM). This technique allows the visualization and characterization of very thin fluid films through transparent substrates such as glass [26]. Second, to investigate the functional effects of a fluid in the contact zone, we compared the adhesive performance of spider setae with that of gecko setae, which are known dry adhesives.

## Results

We found clear evidence of footpad secretions in every species we studied: the spiders *Grammostola rosea* (Theraphosidae, Fig. 1a,b), *Salticus scenicus* (Salticidae, Fig. 1d), and *Cupiennius salei* (Ctenidae, Video S1), the mites *Gromphadorholaelaps schaeferi* (Fig. 1c) and *Balaustium murorum*, and the solifugid *Gluvia dorsalis* (Fig. 1e,f). Detailed characterization of the fluid deposited on glass showed that arachnids left persistent, hydrophobic footprints much like those of insects [5]–[9].

### Fluid Characterization

**Figure 2:**
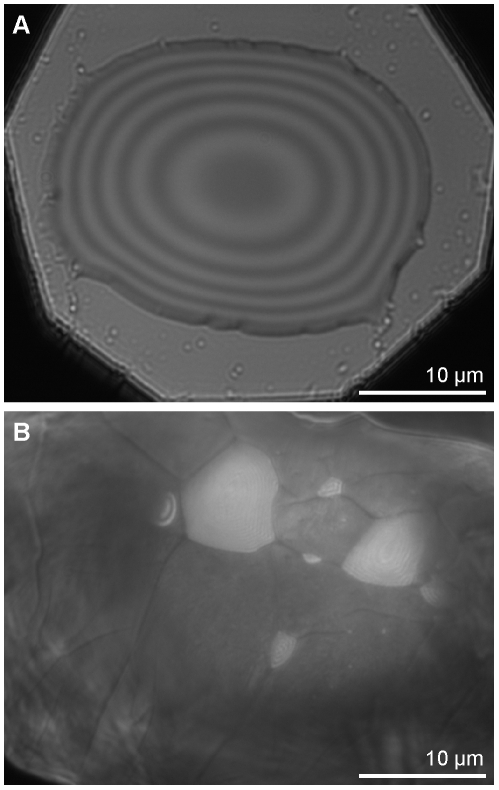
Interference reflection microscopy of footprint secretions. (A) Persistent, hydrophobic tarsal fluid from the tarantula (*Grammostola rosea*) after aggregation of footprint for analysis, as viewed under green light (546 nm). (B) Volatile, hydrophilic droplets trapped under the foot of a mite (*Gromphadorholaelaps schaeferi*) appear lighter than the surrounding pad.

We measured the refractive indices and contact angles of deposited fluid footprints in four of the study species (Table 1). For all of them, the refractive index of the fluid was close to 1.5, similar to previous measurements of hydrophobic insect footprints [5]. Both spider species as well as the mite secreted fluids with contact angles on glass near 10°, while the solifugid fluid had a significantly lower contact angle (4.0°±0.46 SE). Visible fluid footprints and trails were composed of extremely small droplets, ranging from less than one-thousandth of a femtoliter to several femtoliters in volume. Aggregate droplets imaged for analysis had a volume of at most 100 femtoliters (Fig. 2a).

**Table 1:** Properties of arachnid footprint fluids from four species (mean ± SE).

	Contact Angle (°)		Refractive Index	
	fresh	48 h later	fresh	48 h later
**Araneae**				
*Salticus scenicus* (n = 3)	10.3°±0.55	10.1°±0.43	1.49±0.001	1.50±0.002
*Grammostola rosea* (n = 3)	10.3°±0.43	10.5°±0.84	1.50±0.001	1.50±0.001
**Acari**				
*Gromphadorholaelaps schaeferi* (n = 3)	11.2°±0.42	10.2°±0.39	1.49±0.001	1.48±0.001
**Solifugae**				
*Gluvia dorsalis* (n = 3)	4.0°±0.46	3.7°±0.25	1.50±0.001	1.50±0.001

The fluid was very stable and remained on the glass for at least 48 hours without any apparent change in properties. The fact that the secretion remained liquid over multiple days rules out the possibility that we were observing haemolymph from a damaged pad. Exposing the coverslip to water droplets failed to dissolve the fluid, indicating its hydrophobic nature (Video S2).

IRM images of the *Gromphadorholaelaps* mite further showed large hydrophilic droplets trapped between the smooth pad and the glass (Fig. 2b; Video S3), closely resembling the volatile hydrophilic fluid components seen in ants [5] and stick insects [6], [13].

**Figure 3:**
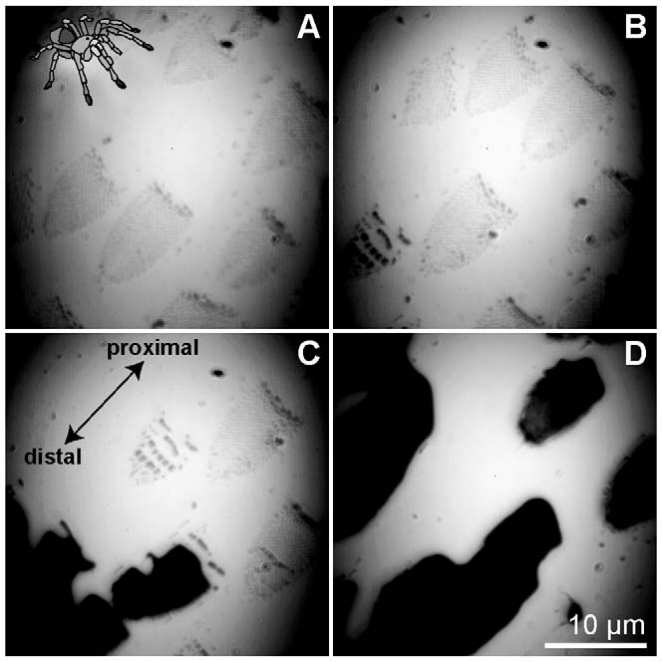
*Grammostola* setae at various stages of wetting. (A) Little or no fluid has accumulated underneath setae, and individual spatulae are still visible. (B) Small fluid droplets, appearing here as dark areas, bridge the gaps between spatulae. (C) A continuous fluid layer forms underneath each seta. (D) Fluid accumulates, bridging gaps between setae. See Video S4 for complete footage.

As the spider *Grammostola* clung inverted to a glass cover slip, fluid first appeared at individual spatulae, bridging the gaps between them, until a continuous layer of fluid formed underneath each seta, finally bridging the gaps between setae (Fig. 3; Video S4). We noted that *Grammostola* setae continued to release fluid even after they were isolated from the animal.

### Single seta force measurements

Fluid had a significant effect on the attachment performance of fibrillar adhesives. Individual setae from the tarantula (*Grammostola rosea*) generated on average a higher shear force (290 µN±30 SE) than setae from the gecko (*Thecadactylus rapicauda*; 41 µN±6.0). Spider setae had more and larger spatulae (1100±57 SE spatulae; approx. area 0.03–0.05 µm^2^) than geckos (480±23 SE spatulae; approx. area 0.02–0.03 µm^2^), but even when normalized by potential contact area (number of spatulae * spatula area), the tarantula setae generated approximately twice as much force.

A one-second pause in the shearing motion did not elicit a significant decrease in force for *Thecadactylus* setae (Table 2), whereas shear force immediately and sharply decreased in *Grammostola* setae after the dragging motion ended, and continued to decrease even further if the pause was extended beyond one second. We observed that dragging an immobilized *Cupiennius salei* spider foot across glass at different rates caused it to release more fluid at higher velocities (Video S3).

**Table 2:** Direct comparison of single seta force in a gecko and a spider (mean ± SE).

	Gecko*Thecadactylus rapicauda*n = 18 setae3 individuals	Spider*Grammostola rosea*n = 18 setae4 individuals
Shear force	41 µN±6.0	290 µN±30
Adhesive force	12 µN±2.0	33 µN±5.1
Remaining friction	96%±1.7	26%±2.8
Remaining adhesion	95%±3.0	12%±5.0
Spatulae per seta	480 spatulae±23*	1100 spatulae±57**

*n = 16 setae from the same experiment.

**n = 25 setae from an independent sample.

## Discussion

We report here the discovery of fluid secretions associated with arachnid adhesive footpads, from diverse arachnid species varying many orders of magnitude in mass. We can only speculate as to why arachnid fluid secretions went unnoticed for so long, but some combination of the following reasons was likely at work: researchers do not expect to see them; the droplets are very small; their refractive index is close to that of glass; and fluids are not continuously secreted. The only previous suggestion in the literature of footprints associated with arachnid adhesive pads was in the mite *Tetranychus urticae* [27], but no evidence was presented for the liquid nature of the footprint, its source or function.

### Properties of Arachnid Tarsal Secretions

The properties of arachnid fluid secretions parallel those of insect footpad secretions, where a persistent hydrophobic fluid is left behind on glass [7], [28]–[30]. There is no existing histological evidence for glands or ducts to synthesize and transport fluid to arachnid adhesive pads [19]–[21], [24], [31], but this was initially the case for insects as well. The positive identification of pores for secretion delivery in insects has only been achieved using transmission electron microscopy, in targeted studies [8], [29]. The exact source of those secretions remains unclear, and multiple glands may be involved [29], [32]. *Grammostola* setae are hollow (pers. obs.) and we observed that they continued to release fluid even after being isolated from the animal. In this species, fluid may flow through the setal stalk, but more work is needed to establish how the fluid ultimately wets individual spatulae.

Volatile hydrophilic droplets appeared trapped between the smooth pad of the *Gromphadorholaelaps* mite and the glass cover slip substrate (Fig. 2b; Video S2), suggesting that the biphasic foot secretions seen in ants and stick insects [5], [13] are present in at least one arachnid, and likely others. The stable hydrophobic components of insect fluid are similar to cuticular lipids [7], [28], but we understand little about the nature of the hydrophilic components.

### Adhesive Performance in Wet vs. Dry Systems

Comparative measurements of adhesive forces from fluid-secreting spider setae and dry gecko setae demonstrate that there are consequences for attachment performance when a fluid is introduced into the footpad-substrate interface. We found that individual *Grammostola* (spider) setae generated more shear and adhesive force per potential contact area than *Thecadactylus* (gecko) setae, and that individual gecko setae maintained higher levels of static friction, whereas spider setae slid relatively easily.

However, this work is preliminary and future investigations need to take into account several additional important factors. First, the calculation of attachment force normalized by contact area assumes that all spatulae make contact, which is not necessarily true. Second, since the spider setae secreted fluid onto the experimental substrate and were then dragged repeatedly across the substrate during each trial, it is likely that those setae were adhering in the presence of accumulated fluid. Accumulation of fluid reduces attachment forces in stick insects [6], and this effect almost certainly accounts for the low static force seen in spiders. The ability to generate static friction has important implications for locomotor control and maneuverability. Without it, the animal would slip frequently, incurring high energetic costs to maintain its position or trajectory. Video footage of a *Grammostola* foot adhering to glass showed a significant amount of fluid developing over time as the animal attempts to remain attached, upside-down, to the cover slip substrate (Fig. 3; Video S4). It is unlikely that, in natural conditions, spiders routinely experience fluid accumulation to the degree we observed, and our shear and adhesive force values for *Grammostola* may in fact represent a lower bound on their performance capability.

Finally, our observations suggest that spiders may control fluid release, either actively or passively, limiting the application of their secretion to high-velocity situations (Video S1). If spiders are more likely than geckos to experience low static forces due to accumulation of fluid, it would be advantageous for them to avoid secreting fluid during slow movements and instead adhere via dry intermolecular forces as the gecko does. Similarities in their morphology suggest that this is possible [22], and we plan to investigate this hypothesis with future studies.

### Theraphosid Tarsal Silk

**Figure 4:**
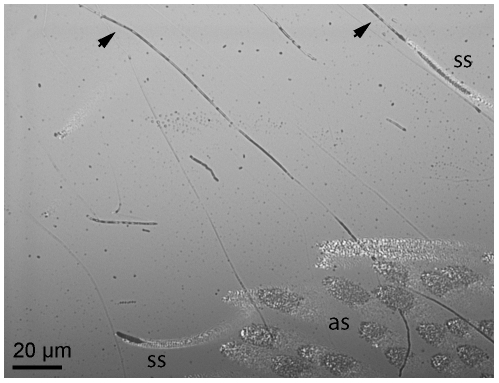
Evidence for tarsal silk in the tarantula *Grammostola rosea*. Silk strands are indicated with arrows. Adhesive setae (as; lower right) far outnumber silk-producing setae (ss) on the tarsus. Dark areas indicate fluid or silk secretion; bright areas indicate thin layers of air between the setae and the glass surface. See Video S5 for complete time-lapse video footage of silk being secreted.

Previous investigators observed a silk-like material secreted from the tarsi of the theraphosid *Aphonopelma seemanni* [25], and this observation was later questioned [33]. We can confirm that occasional strands of a silk-like substance were exuded from the feet of *Grammostola rosea*, another theraphosid (Fig. 4; Video S5). We did not observe tarsal silk or the associated silk-producing setae in the jumping spider. Silk-producing setae on *Grammostola* tarsi were substantially outnumbered by adhesive setae (ca. 50 adhesive setae per silk-producing seta), and the silk did not appear to sustain a tensile load (strands were not always stretched taut, and often broke). We consider it unlikely that these silk-like secretions contribute significantly to attachment force. It remains an open and interesting question how the evolution and development of these setae might be related to abdominal spinnerets.

### Conclusions

Arachnids remain a large, and largely understudied, group of organisms from which we can draw biological and technical insights into the general principles of adhesion. From an evolutionary standpoint it is clear that tarsal secretions are widespread among arthropods; such secretions can have important ecological consequences, for instance in chemical communication [34]. Spiders represent the compelling possibility of a hybrid fibrillar adhesive that takes advantage of both wet and dry adhesive mechanisms. Whereas previous studies assumed a dry adhesive system and interpreted the results as such, future investigators will be able to take a more comprehensive approach toward arachnid adhesive function.

## Materials and Methods

### Study organisms

*Grammostola rosea* spiders were obtained from a pet supplier (Coast to Coast Exotics, Darlington, UK). *Salticus scenicus* spiders and *Balaustium murorum* mites were collected in Cambridge, UK. *Gromphadorholaelaps schaeferi* mites were collected from live Madagascar hissing roaches kept in our laboratory in the Dept. of Zoology, University of Cambridge, UK. *Gluvia dorsalis* solifugids were collected in Évora, Portugal. *Cupiennius salei* spiders were provided by Dr. Friedrich Barth from his laboratory colony in the Dept. of Neurobiology, University of Vienna, Austria. All spiders and solifugids were individually housed and fed on a diet of crickets and water. Mites were re-released after the experiments ended.

*Thecadactylus rapicauda* geckos were obtained from California Zoological Supply (Santa Ana, CA, USA) and cared for by the Office of Laboratory Animal Care at the University of California, Berkeley, where they were fed on a diet of crickets and water.

### Ethics statement

All experiments involving arachnids were performed in the Zoology Dept. at the University of Cambridge, in accordance with the Animals (Scientific Procedures) Act of 1986. Experiments involving *Thecadactylus* were conducted in the Dept. of Integrative Biology at the University of California Berkeley, in accordance with Animal Use Protocol R137 (as approved by the Animal Care and Use Committee).

### Fluid Characterization

We viewed three live specimens each from two species of spider (Theraphosidae: *Grammostola rosea*, Salticidae: *Salticus scenicus*), one mite (Laelapidae: *Gromphadorholaelaps schaeferi*), and one solifugid (Daesiidae: *Gluvia dorsalis*) attaching to the underside of a glass cover slip with an upright microscope. We filmed movies of the contact using a high-speed HotShot PCI 1280 B/W digital video camera (NAC Image Technology, Simi Valley, CA, USA), and a 10-bit B/W QICAM digital camera (QImaging, Surrey, BC, Canada). In the event that animals were not able or motivated to remain attached while inverted to the coverslip for the duration of imaging, we allowed them to climb across an inclined coverslip for a sustained period of time before imaging the fluid left behind.

Interference reflection microscopy (IRM) allowed us to measure the refractive index, contact angle and volume of fluid left behind on the glass. Fluid droplets as deposited by the animals were too small to conduct IRM measurements, so we aggregated multiple droplets from each footprint by dragging a fine glass rod with spherical tip across the surface (Fig. 2a). Images of ten droplets per individual (three individuals per species) were taken with green (546 nm) epi-illumination using a Leica DRM HC series microscope (Leica Microsystems, Wetzlar, Germany) and the 10-bit B/W QICAM camera. We then used intensity line-plots to measure the relative contrast of adjacent interference extremes. To calculate the refractive indices of the deposited droplets, the contrasts of the interference fringes [(I_max_−I_min_)/(I_max_+I_min_)] were compared with the contrasts from similar-sized droplets of calibration fluids (water-glycerol mixtures and immersion oil) with known refractive indices. As the interference patterns of water-glycerol droplets with steeper gradients are damped by the optical resolution of the microscope [26], only droplet sections with contact angles comparable to those of footprint droplets were used for analyzing fringe contrasts. Contact angles of footprint droplets were also measured from the intensity line-plots. A discussion of the technique as applied to insect adhesion can be found in [5]. This process was repeated after 48 hours to confirm that the droplets were still fluid and to discover if their properties or volume changed over long timescales.

To determine whether the footprint secretions were hydrophobic or hydrophilic, we deposited small water droplets (5–30 µm in diameter) onto the glass cover slip using an ultrasonic humidifier (Honeywell, BH-860 E) and observed whether the footprint droplets dissolved into them.

### Single seta force measurements

Individual setae from the claw tufts and tarsi of *Grammostola rosea* (N = 18 setae; 4 individuals) were harvested from living animals and mounted to insect pins using 5-minute epoxy (Bondloc UK Ltd, Bewdley, UK). Each pin was clamped into a pin vice and fixed to a three-dimensional DC motor stage (M-126PD, Physik Instrumente, Karlsruhe, Germany), which drove the seta through pre-programmed shearing motions 0.5–2 mm in amplitude, at set velocities (0.5–4 mm/s). Setae were dragged across a smooth glass substrate glued to the tip of a 58.9 mm length of tungsten wire with a 0.1 mm radius. The spring constant of this bending beam was determined to be 0.46 N/m. The movement of the beam was captured using a Redlake PCI 1000 B/W high-speed video camera (Redlake, Tallahassee, FL, USA), and digitized using ProAnalyst Lite (Xcitex Inc, Cambridge, MA, USA) to yield peak shear and adhesive force, remaining shear and adhesive force after a one-second pause, and the velocity of the seta.

An analogous method was used to measure single seta forces in the gecko *Thecadactylus rapicauda*, using a smooth silicon substrate fixed to a 46 mm steel wire with radius 0.06 mm and spring constant of 0.079 N/m, at velocities between 0.4–2.8 mm/s (0.5–2 mm sliding distance). Details can be found in [35].

## Supporting Information

Video S1 ***Cupiennius salei*** **seta sliding across glass.** As the seta is dragged across glass, it deposits fluid. More fluid appears to be deposited during fast movements than during slow ones.
(MOV)

Video S2 ***Grammostola rosea*** **fluid before and after exposure to water vapor.** The secretion does not dissolve in water, indicating its hydrophobic nature.
(MOV)

Video S3 **Evidence for a biphasic fluid in arachnids.** Hydrophilic droplets underneath the foot of the mite *Gromphadorholaelaps schaeferi* evaporate quickly once they reach the edge of the pad, much like the volatile hydrophilic component of biphasic adhesive secretions seen in insects.
(MOV)

Video S4 ***Grammostola*** **setae at various stages of wetting.** Close up of *Grammostola rosea* setae as the animal clings to the underside of the glass. Dark areas are continuous layers of trapped fluid. Camera initially shows distal setae completely covered in fluid, then scans proximally along claw tuft to show setae where less fluid has accumulated and individual spatulae are still visible.
(MOV)

Video S5 **Time lapse video of a** ***Grammostola rosea*** **foot sliding across glass.** Adhesive setae leave behind clusters of minute fluid droplets, while other setae secrete silk.
(MOV)
